# HPV-16, HPV-58, and HPV-33 are the most carcinogenic HPV genotypes in Southwestern China and their viral loads are associated with severity of premalignant lesions in the cervix

**DOI:** 10.1186/s12985-018-1003-x

**Published:** 2018-05-25

**Authors:** Wenbo Long, Zixi Yang, Xiabin Li, Ming Chen, Jie Liu, Yuanxue Zhang, Xingwang Sun

**Affiliations:** Pathology Department of the First Affiliated Hospital, Southwest Medical University, Taiping Street No.25, Jiangyang District, Luzhou City, Sichuan China

**Keywords:** Correlation analysis, High-risk HPV subtype, Human Papillomavirus (HPV), Intraepithelial lesion, Viral load

## Abstract

**Background:**

Currently, the role of human papillomavirus (HPV)-58 in southwestern China has been unexplored. Although there is some controversy, it is proposed that the viral load of HPV correlates with the severity of intraepithelial lesions.

**Methods:**

We identified 7747 patients from south Sichuan and adjacent regions who were diagnosed with HPV between 2013 and 2017. The HR-HPV subtype distribution was analyzed and the patient’s viral loads were quantified using real-time RT-PCR.

**Results:**

Among all 7747 patients screened for HPV genotypes, 1728 patients (22.31%) were identified as having HR-HPV subtypes. In patients without intraepithelial lesions (12.41%), HPV-52, HPV-16, and HPV-58 were the three most prevalent HR-HPV subtypes. Moreover, HPV-16, HPV-58, and HPV-33 were the most prevalent subtypes in patients with cervical intraepithelial neoplasia grade II (CINII) (42.86%) and grade III (CINIII) (59.81%), and accounted for the majority of invasive cervical cancer (ICC) (69.34%). Thus, viral loads of HPV-58, HPV-16, and HPV-33 positively correlated with the severity of cervical lesions (*P* < 0.001, *P* = 0.016, *P* = 0.026, respectively). Using receiver operating characteristic (ROC) curve analysis, the optimum thresholds for predicting severe intraepithelial lesions of cases (CINI, CINIII and ICC) with HPV-16, HPV-58, and HPV-33, respectively, were obtained, which were 1, 0.93, and 0.25, respectively.

**Conclusion:**

In our study, we showed that HPV-16 was the most common carcinogenic HPV subtype in southwestern China followed by HPV-58 and HPV-33. Viral loads of these subtypes are associated with the severity of premalignant lesions in the cervix.

**Electronic supplementary material:**

The online version of this article (10.1186/s12985-018-1003-x) contains supplementary material, which is available to authorized users.

## Background

Human Papillomaviruses (HPVs) belong to the highly heterogeneous family of DNA viruses, and can cause intraepithelial neoplasias in skin and mucosal cells. Roughly 40 high-risk (HR)-HPV types are found in the female genital tract, which have the potential to drive the evolution of high-grade premalignant lesions into cervical carcinomas [1]. Thus, for optimizing cervical carcinoma preventive strategies, the prevalence of HR-HPV subtypes in women in certain areas should be further explored.

The distribution of HPV types varies geographically. In a previous report, covering millions of cases from five continents, it was indicated that HPV-16 was the most prevalent and carcinogenic genotype worldwide, followed by HPV-18 in many regions [2]. However, HPV-31 and 33 rank second in Brazil and HPV-52 ranks second in Africa [2, 3], indicating the importance of non-HPV-16/18 subtypes in certain regions. According to a study in 13 cities in Korea, the three most common HR-HPV types in patients with intraepithelial lesions are HPV-52, 58, and 16 [4]. These findings were similar to studies performed in populations in southeast China [5], southern Taiwan [6], and Japan [7], suggesting a higher prevalence of HPV-58 and HPV-52 in women with cervical intraepithelial neoplasia in Asian countries.

Integration of HR-HPV into the host genome is vital for malignant transformation [8, 9]. Therefore, a high load of HPV DNA may increase the chances of malignancy. The load of viral DNA has been reported to be associated with the risk of dysplasia and carcinoma [10, 11], suggesting this may serve as a quantitative method to screen for precancerous cervical lesions. However, the relationship between HPV DNA load and the severity of cervical lesions is still controversial. For example, the results of a study performed by Lorincz et al. [12] indicated that a high viral load of 13 carcinogenic HPV types did not predict the risk of CINIII or worse, and the data presented by Wu et al. [13] showed that HPV-18 viral load was low in precancerous cases but increased in cancer. In this study, we carried out an intensive investigation to uncover HPV genotype distribution according to histopathological diagnosis and analyzed the correlation of viral load and severity of intraepithelial lesions. We aimed to explore whether viral load could be employed to predict the likelihood of cervical cancer for patients with precancerous lesions.

## Methods

### Clinical specimen collection

A total of 7747 specimens from patients in southern Sichuan and the adjacent areas were collected between January 2013 and November 2017 in the First Affiliated Hospital of Southwest Medical University, China. The majority of the patients showed lesions whereas the remainder visited the hospital for routine cervical exams. The age of the patients ranged from 16 to 86 years with an average of 40.33 ± 9.36 years. All patients first underwent routine cytological screening (thinprep cytological test, TCT). Patients with intraepithelial lesions, malignancy or who showed other morphologic abnormalities by TCT, or who had a HPV infection underwent histological (biopsy) and histopathological procedures, then a final diagnosis was made. The results of each procedure were interpreted by two experts, and if they did not agree on a diagnosis, a third expert was consulted to finalize the diagnosis. Patients with malignancies outside the reproductive system were excluded from this study.

### DNA preparation

For the HPV test, cervical samples were obtained by gynecologists via a cytobrush, and resuspended in 20 mL of liquid-based cytology medium. To extract DNA from these samples, 50 μL of the liquid-based cytology sample was pelleted, and 200 μL of the denaturing reagent (Tellgen Life Science Co.Ltd. Shanghai, China) was added to the pellet. Then, samples were incubated at 100 °C for 10 min, and centrifuged for 5 min at 10,000 RPM. Supernatant was collected and the DNA concentration was measured using a NanoDrop 2000 spectrophotometer.

### PCR amplification

Cervical specimens were examined for HPV DNA using a Slan-96P Real time PCR Systemassay (Hongshi medical technology Co. Ltd. Shanghai, China). The following 13HR-HPV genotypes were evaluated: HPV-16, 18, 31, 33, 35, 39, 45, 51, 52, 56, 58, 59, and 68. The PCR program was performed as described previously [14]. The dual specificity tyrosine phosphorylation regulated kinase 1A (DYRK1a) gene served as a reference gene. Viral load was calculated according to the following formula: viral load = 2 ^ (reference CT – objective CT). CT: cycle threshold.

### Cytological and pathological diagnosis

Classifications of lesions in TCT were performed in conformity with the Bethesda 2001 criteria, including negative for intraepithelial lesion or malignancy (NILM), which includes normal and inflammatory tissues; atypical squamous cells of undetermined significance/cannot exclude high grade lesion (ASC-US/H); low grade squamous intraepithelial lesion (LSIL); high grade squamous intraepithelial lesion (HSIL); cervical squamous cell carcinoma (SCC); atypical glandular cells (AGC); endocervical adenocarcinoma (ECA).

Patients with intraepithelial lesions or who showed other morphologic abnormalities by TCT, or had a HPV infection underwent a pathological procedure for final diagnosis. The biopsy specimens obtained were fixed in formalin, embedded in paraffin, and stained with hematoxylin-eosin. Pathological identifications were performed in conformity with the 2014 World Health Organization (WHO) (Fourth Edition) classification criteria and described as follows: a normal cervix describes those who are negative for intraepithelial lesions or malignancy and do not have inflammation or other benign lesions. Inflammation describes those with inflammatory or other benign lesions, including chronic cervicitis, cervical hypertrophy, nabothian cysts, erosion, bleeding, and hyperplasia. CINI describes cervical intraepithelial neoplasia grade I (low dysplasia), corresponding to LSIL. CINII and CINIII describe cervical intraepithelial neoplasia grade II (moderate dysplasia) and III (severe dysplasia), respectively, corresponding to HSIL. ICC describes invasive cervical cancer, whereas others describe cases after surgery or treatment of hysteromyoma, and so on.

### Statistical analysis

Analyses were carried out using SPSS software. A Pearson’s χ^2^ test was performed to evaluate the significance of differences between designated groups. All analyses were two-sided. Pearson’s correlation analysis was employed to evaluate the relationship between viral load and the severity of intraepithelial lesions. In the analysis, CINI, CINII, and CINIII cases were given a score of 1, 2, and 3, respectively, cancer cases were given a score of 4, whereas others without definite intraepithelial lesions were given a score of 0. ROC curves were calculated to indicate the optimum thresholds for predicting intraepithelial lesions and severe intraepithelial lesions in cases with HPV-16, HPV-58, and HPV-33 subtypes.

## Results

### HPV infection in the study cases

Of the samples obtained, 6735 (86.94%) cases were negative for intraepithelial lesions (either the patients had a normal cervix or they presented with inflammation), 618 (7.98%) cases had intraepithelial lesions or cancer, and 394 (5.09%) cases showed other characteristics (Additional file 1: Table S1 and Table 1). All 7747 patients were screened for HPV genotypes and 1728 (22.31%) of patients were identified as having HR-HPV subtypes. The HR-HPV infection rate in patients with non-intraepithelial lesions was 18.89%, while the infection rates in patients with CINI, CINII, CINIII, and ICC cases were 48.37, 61.34, 70.81, and 78.67%, respectively. The HPV subtype was most prevalent in the > 55 age patient group with a detection rate of 33%. Single HPV subtype infections were detected in 80.45 and 79.32% of patients with a normal cervix and inflammation, respectively, were lower in CINI, CINII, and CINIII patients (73.33, 73.97, and 74.83%, respectively), and higher in ICC patients (86.44%).

**Table 1:** Distribution of the 13 High-risk Human papillomavirus (HR-HPV) infection according to final diagnoses among the 7747 patients

Diagnosis	Infected (%)	Negative (%)	Total	χ^2^value	*P* value
Normal cervix	628 (19.84)	2537 (80.16)	3165	/	/
Inflammation	644 (18.04)	2926 (81.96)	3570	3.56	0.169
CINI	104 (48.37)	111 (51.63)	215	96.58^**^	< 0.001
CINII	73 (61.34)	46 (38.66)	119	117.66^**^	< 0.001
CINIII	148 (70.81)	61 (29.19)	209	287.62^**^	< 0.001
ICC	59 (78.67)	16 (21.33)	75	151.74^**^	< 0.001
Others	72 (18.27)	322 (81.73)	394	0.55	0.761
Total	1728 (22.31)	6019 (77.69)	7747	8.05^*^	0.018

Normal cervix is served as test control. Normal cervix describes those who are negative for intraepithelial lesions or malignancy and do not have inflammation or other benign lesions; Inflammation describes those with inflammatory or other benign lesions, including chronic cervicitis, cervical hypertrophy, nabothian cysts, erosion, bleeding, and hyperplasia. CINI describes cervical intraepithelial neoplasia grade I (low dysplasia), corresponding to LSIL. CINII and CINIII describe cervical intraepithelial neoplasia grade II (moderate dysplasia) and III (severe dysplasia), respectively, corresponding to HSIL. ICC describes invasive cervical cancer, whereas others describe cases after surgery or treatment of hysteromyoma, and so on

^**^The significance is at the *P* ≤ 0.01 level

^*^The significance is at the *P* ≤ 0.05 level

### The HPV genotype distribution according to final diagnostic status

The distribution of HPV genotypes relative to the severity of cervical lesions was investigated and the data is presented in Table 2. In patients without intraepithelial lesions, the three most prevalent genotypes were HPV-52 (5.75%, 387/6735), HPV-58 (3.36%, 226/6735), and HPV-16 (3.31%, 223/6735). In contrast, the most common HPV genotypes were HPV-58, HPV-16, and HPV-52 in patients with CINI and CINII, and HPV-16 and HPV-58in patients with CINIII and ICC.

**Table 2:** Distribution of the 13 High-risk Human papillomavirus (HR-HPV) genotypes according to final diagnoses among the 1728 HPV infected patients

Genotype	Normal cervix	Inflammation	CINI	CINII	CINIII	ICC	Others	Total	Rate (%)	χ^2^ value	*P* value
HPV-56	39	38	8	1	3	1	8	98	4.38	/	/
HPV-16	119	104	22	20	78	41	12	396	17.69	36.91^**^	< 0.001
HPV-18	34	36	5	2	5	5	6	93	4.15	4.74	0.691
HPV-31	24	37	3	2	10	1	6	83	3.71	9.07	0.248
HPV-33	34	29	5	14	20	4	8	114	5.09	26.82^**^	< 0.001
HPV-35	31	30	4	3	7	0	8	83	3.71	5.58	0.589
HPV-39	56	54	10	3	3	1	8	135	6.03	1.20	0.991
HPV-45	15	13	3	1	1	0	2	35	1.56	1.23	0.990
HPV-51	60	65	11	7	2	1	2	148	6.61	10.58	0.158
HPV-52	186	201	29	20	16	2	18	472	21.08	7.01	0.427
HPV-58	90	136	31	19	42	10	14	342	15.27	18.22^**^	0.009
HPV-59	36	46	6	2	5	1	5	101	4.51	2.65	0.915
HPV-68	60	58	7	4	3	1	6	139	6.21	3.79	0.803

^**^The significance is at the *P* ≤ 0.01 level. The data of HPV-56 is employed as test control

A total of four α-papillomavirus species were analyzed in this study, including α-5 (HPV-51), α-6 (HPV-56), α-7 (HPV-18, 39, 45, 59, and 68), and α-9 (HPV-16, 31, 33,35, 52, and 58). The HPV infection rates of α-5, α-6, and α-7 in patients were much lower compared to that of α-9. Cases with α-9 infection accounted for 13.78, 13.05, 36.74, 76.47, 68.42, and 72% in patients with no symptoms, inflammation, CINI, CINII, CINIII, and ICC, respectively.

**Fig. 1:**
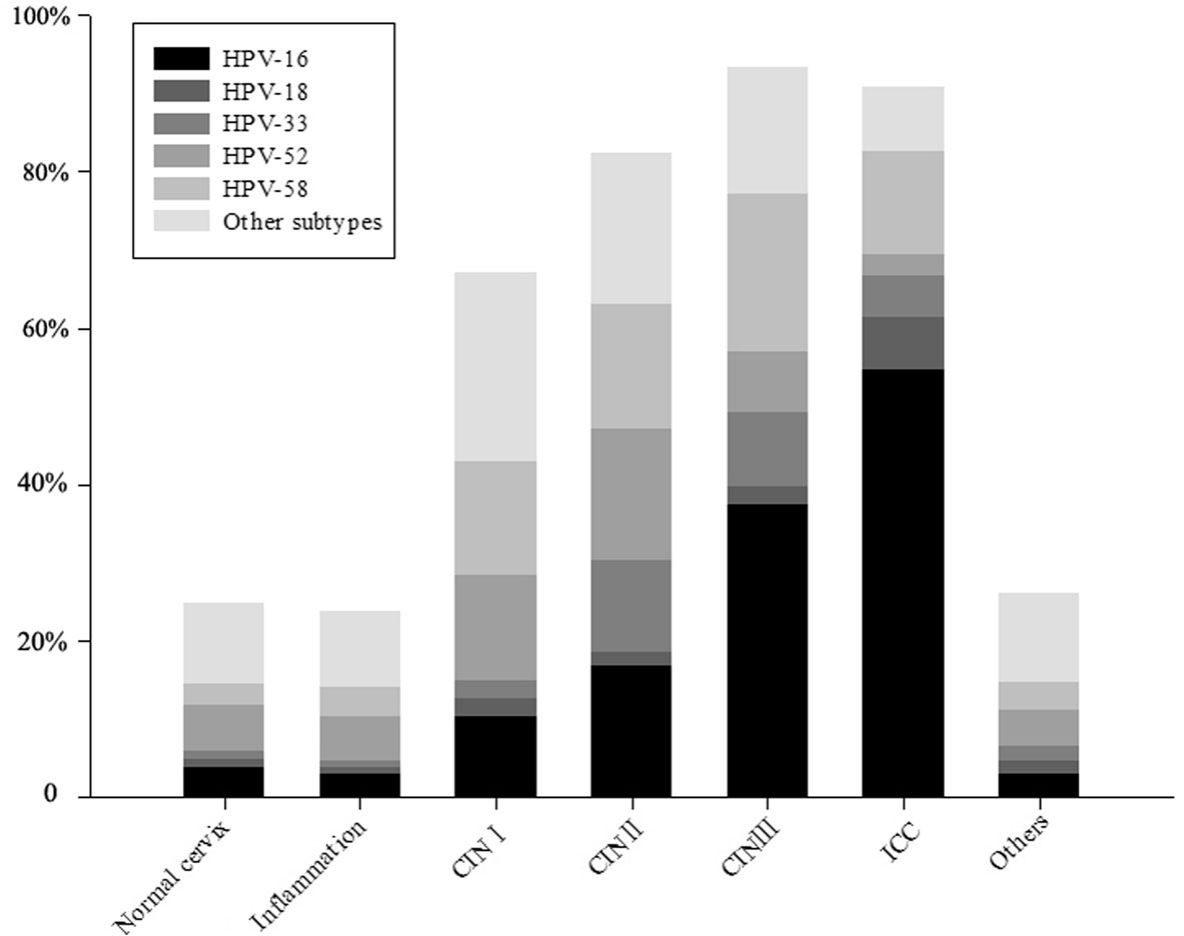
Distribution of the five most prevalent Human papillomavirus (HPV) subtypes according to diagnosis

HPV-52 was the most prevalent subtype in 7747 cases, however its prevalence decreased with the severity of intraepithelial lesions. By Pearson’s χ2 test, HPV-16, HPV-58, andHPV-33 accounted for the majority of cases with severe cervical lesions (*P* < 0.01), and accounted for 25.12, 42.86, 59.81, and 69.34% in CINI, CINII, CINIII, and ICC patients, respectively (Fig. 1). The majority of cervical cancer was attributed to HPV-16 (54.67%), followed by HPV-58 (13.33%), and HPV-33 (5.33%).HPV-16 also accounted for most HSIL (CINII and CINIII) with a rate of 16.81 and 37.32%, respectively, followed by HPV-58 (15.97 and 20.1%). Interestingly, HPV-16 was only the third prevalent genotype found in CINI.

### Correlation analysis to study the relationship between HPV DNA load and patient age and severity of intraepithelial lesions

In general, no significant correlation was observed between the total viral load and the age of patients (*P* = 0.714) (Table 3). For specific HPV genotypes, patient age positively correlated with the viral load of HPV-58 (*P* = 0.027) and negatively correlated to the viral load of HPV-59 (*P* = 0.022). The severity of the cervical lesions positively correlated to the viral load (*P* < 0.001). For specific genotypes, viral loads of HPV-16, HPV-58, and HPV-33 significantly correlated with the severity of intraepithelial lesions, with correlation coefficients of 0.121 (*P* = 0.016), 0.189 (P < 0.001), and 0.209 (*P* = 0.026), respectively. The viral loads of other subtypes did not significantly correlate with the severity of lesions.

**Table 3:** Correlation analyses of the relationships between HPV DNA load and patient age and the severity of intraepithelial lesions

Genotype	CC with patient age	*P* value	CC with lesion severity	*P* value
HPV-16	0.041	0.425	0.121^*^	0.016
HPV-18	−0.182	0.08	−0.104	0.322
HPV-31	−0.041	0.715	0.071	0.523
HPV-33	0.003	0.978	0.209^*^	0.026
HPV-35	−0.143	0.198	−0.025	0.822
HPV-39	− 0.167	0.053	0.026	0.768
HPV-45	−0.025	0.886	− 0.083	0.637
HPV-51	−0.14	0.868	0.124	0.134
HPV-52	−0.019	0.674	0.088	0.057
HPV-56	0.22	0.03	0.025	0.804
HPV-58	0.102^*^	0.027	0.189^**^	< 0.001
HPV-59	−0.228^*^	0.022	−0.068	0.5
HPV-68	−0.032	0.707	−0.032	0.706
Total HPV	−0.008	0.714	0.143^**^	< 0.001

*CC* Correlation coefficient

^**^The significance is at the *P* ≤ 0.01 level

^*^The significance is at the *P* ≤ 0.05 level

**Fig. 2:**
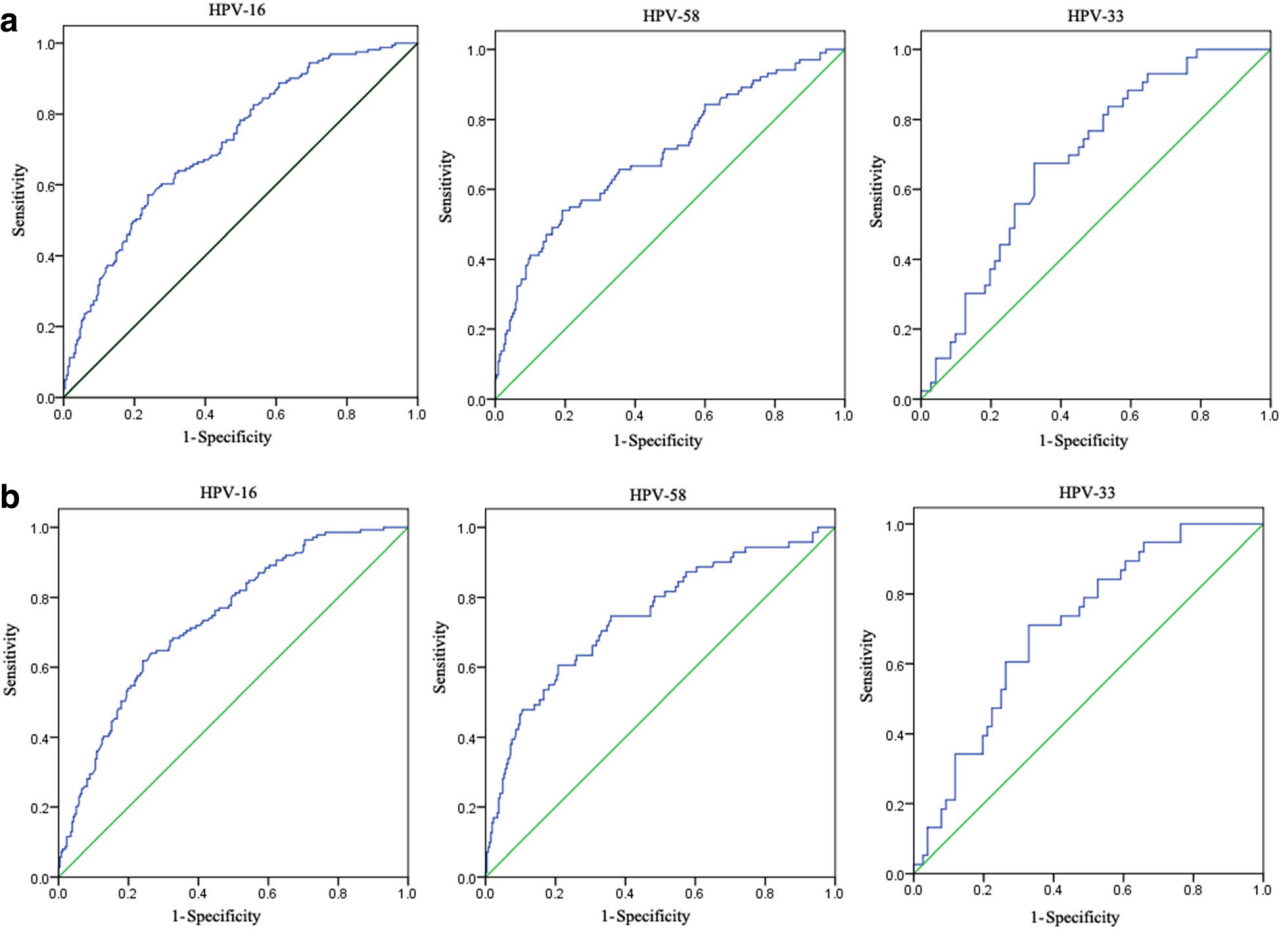
**a** ROC curves for viral loads and intraepithelial lesions (CINI-III, and ICC); **b** ROC curves for viral loads and severe intraepithelial lesions (CINII-CINIII, and ICC)

To quantitatively evaluate intraepithelial lesions (CINI-CINIII, and ICC), the ROC curves for viral loads and intraepithelial lesions in cases with HPV-16, HPV-58, and HPV-33 were calculated (Fig. 2a). Optimum thresholds for predicting intraepithelial lesions in cases with HPV-16, HPV-58, and HPV-33 were 1, 0.7, and 0.25, respectively. In cases with HPV-16, sensitivity, specificity, positive predictive rate, and negative predictive rate for predicting intraepithelial lesions were 0.669, 0.604, 0.535, and 0.724, respectively. Moreover, in cases with HPV-58they were 0.667, 0.613, 0.422, and 0.812, respectively, and in cases with HPV-33 they were 0.674, 0.662, 0.558, and 0.774, respectively, Considering that severe neoplasia was more closely related to cervical cancer, the ROC curves for viral loads and severe intraepithelial lesions (CINII-CINIII, and ICC) were calculated (Fig. 2b). The optimum thresholds for predicting severe intraepithelial lesions in cases withHPV-16, HPV-58, and HPV-33 were 1, 0.93, and 0.25, respectively. Sensitivity, specificity, positive predictive rate, and negative predictive rate for predicting severe intraepithelial lesions were 0.719, 0.607, 0.495, and 0.796, respectively, in cases with HPV-16; were 0.746, 0.623, 0.349, and 0.905, respectively, in cases with HPV-58; and were 0.711, 0.663, 0.519, and 0.823, respectively, in cases with HPV-33.

## Discussion

The correlation between HPV subtype and cervical pathology status presented in this study may supply guidance for HPV vaccination programs and preventative strategies. The overall HR-HPV prevalence among the 7747 patients was 1728 (22.31%), which was similar to the findings of a previous meta-analysis of the Chinese population [15]. The three most prevalent HPV subtypes were HPV-52, HPV-16, and HPV-58, which supported the viewpoint that HPV-52 and HPV-58 accounted for a high ratio among individuals in eastern Asia [4, 7, 15]. In our study, we found that the overall HR-HPV prevalence among ICC patients was 78.67%, which was close to the HPV (both HR-HPV and LR-HPV) prevalence in squamous-cell cervical cancers among 9 countries, ranging from about 80–98% [16].

HR-HPV prevalence rates increased with the severity of the intraepithelial lesions [17–19]. However, only the HPV-16 subtype rate significantly increased with the severity of intraepithelial lesions. In addition, we found that HPV-16 was the most common subtype in both CINIII and ICC cases, suggesting that HPV-16 is also the most carcinogenic in the southern Sichuan of China [20]. Comparatively, although HPV-18 has been demonstrated the second most common genotype worldwide [2, 21], its prevalence in this study was low, only 2.33% in CINI, 1.68% in CINII, 2.39% in CINIII, and 6.67% in ICC patients. In a previous report, it was indicated that HPV-16 associated with both squamous-cell carcinoma and adenocarcinoma of the cervix and that HPV-18 highly correlated with adenocarcinoma [22]. The most common type of ICC identified in the patients included in our study was squamous-cell carcinoma (92%, 69/75), therefore the prevalence of HPV-18 was lower compared to that presented in other reports [5, 20, 23].

HPV-52 has been reported as the most prevalent genotype in many regions, including eastern China [4, 6, 17], and it was one of the main contributors to cervical cancer. However, in our 7747 patients, HPV-52 was the most prevalent HPV subtype in the normal cervix and in patients who presented with inflammation, but the second most common in CINI (13.49%) and CINII (16.81%), the fourth most common in CINIII (7.66%), and the fifth most common in ICC (2.67%). In contrast, HPV-58 was the third most prevalent in non-intraepithelial lesion cases, the most common in CINI (14.42%), the third most common in CINII (15.97%), and the second most common in both CINIII (20.10%) and ICC (13.33%). These findings indicated that HPV-52 was more prevalent in patients with no cervical lesions and LSIL, whereas HPV-58 was more prevalent in patients with HSIL and ICC, which was in accordance with a study of HPV subtype distribution among 40,311 women in southwest China [20]. Another meta-analysis in Korea indicated that HPV-58 was a more prominent subtype in patients with HSIL and ICC cases when compared to HPV-52 [24], which was similar to the findings of a large case-control study on cervical cancer patients in Japan [7]. Thus, the results of our study indicated HPV-58 as the most carcinogenic subtype except HPV-16 in certain regions.

Viral load determined by quantitative methods has been performed to predict the development of high-grade cervical lesions [13, 25, 26]. Whether viral load can be used as a marker to predict cervical neoplasia is currently controversial [12, 27]. In this study, we observed a significant correlation between viral load and the severity of cervical lesions (*P* < 0.001), which was in line with the results described by Dalstein et al. [11]. A majority of HPV infections will not lead to cancer, because a persistent infection is essential for conversion of a low grade lesion to a high grade lesion or cancer [28]. The clearance of HR-HPV was easy in low-load HPV-infected patients, but harder in high-load HPV-infected patients [29]. This may explain why patients with a high viral load tend to develop more severe cervical lesions. Our study supported the viewpoint by demonstrating that more severe intraepithelial lesions were associated with higher viral loads. However, when analyzing specific HPV genotypes, only HPV-16, HPV-58, and HPV-33 loads increased with the severity of lesions, indicating that these three subtypes were the major contributors to cervical cancer by persistent infection. HPV-16 was the most carcinogenic subtype and its viral load in cervical smears has been linked to an elevated risk of future ICC, however this was not the case for HPV-18 or HPV-31 [30]. Our results confirmed the most carcinogenic status of HPV-16 in southwestern China, followed by HPV-58, and HPV-33.

Hildesheim et al. [31] found a threshold value of 10 pg/mL of viral load, above which HR-HPV persisted. Nevertheless, although a linear increase in the HPV viral load with histological grade from normal to cervical cancer has been reported [13], a quantitative method to predict the occurrence of neoplasia cases did not exist. In this study, we established a quantitative method by analyzing the ROC curves for viral loads and intraepithelial lesions. Using the method, optimum thresholds for predicting severe intraepithelial lesions (CINII-CINIII, and ICC) were obtained in cases with HPV-16, HPV-58, and HPV-33 (1, 0.93, and 0.25, respectively). Above these thresholds, 71.9, 74.6, and 71.1% of cases with HPV-16, HPV-58 and HPV-33, respectively, indicated true cases with severe neoplasia or ICC. In addition, under these thresholds, 79.59, 90.53 and 82.26% of cases with HPV-16, HPV-58 and HPV-33, respectively, were true cases with normal cervix, inflammation, or CINI. Given that the sensitivity of cytology to detect CIN is only about 60–80% [11, 32], this method may provide a cheap and practical alternative way to predict cervical lesions by real-time RT-PCR. Nevertheless, the sensitivity and specificity of the approach deserves further improvement, and additional studies should need to be employed to evaluate its clinical use.

## Conclusion

In this study, a broad summary is presented of the distribution of the HPV type in the southern Sichuan province in women with cervical lesions. The most prevalent HPV types in cases with severe cervical lesions were HPV-16, HPV-58, and HPV-33. HPV-16 was the most common carcinogenic HPV type in southwestern China, followed by HPV-58. The viral load of HPV-58 subtype correlated with the severity of cervical lesions, as was the case for HPV-16, and HPV-33. Additional intensive studies are needed to fully evaluate the carcinogenicity of HPV-58 for HPV prevention in southern Sichuan and adjacent areas in China.

## Additional file

Additional file 1: **Table S1.** Original data of final diagnoses and quantitative PCR identification. (XLS 902 kb)

## Abbreviations

CINI: Cervical intraepithelial neoplasia grade I (low dysplasia), corresponding to LSIL
CINII and CINIII: Cervical intraepithelial neoplasia grade II (moderate dysplasia) and III (severe dysplasia), respectively, corresponding to HSIL
CT: Cycle threshold
HPV: Human papillomavirus
HR-HPV: High-risk-Human papillomavirus
HSIL: High-grade squamous intra epithelial lesion
ICC: Invasive cervical cancer
Inflammation: With inflammatory or other benign lesions including chronic cervicitis, cervical hypertrophy, nabothian cysts, erosion, bleeding, and hyperplasia
LSIL: Low-grade squamous intraepithelial lesion
NILM: Negative for intraepithelial lesion or malignancy
Normal cervix: Negative for intraepithelial lesion or malignancy and do not have inflammation or other benign lesions
TCT: Thinprep cytological test
